# Red Cell Distribution Width as an Independent Marker of Objective Disease Activity in Ulcerative Colitis and Crohn’s Disease: A Large Real-World Cohort of 1000 Patients

**DOI:** 10.5152/tjg.2026.25788

**Published:** 2026-04-08

**Authors:** Yavuz Özden

**Affiliations:** Department of Gastroenterology, Kayseri City Hospital, University of Health Sciences, Kayseri, Türkiye

**Keywords:** Crohn disease, diagnostic biomarkers, disease activity, erythrocyte indices, inflammatory bowel diseases, ulcerative colitis

## Abstract

**Background/Aims:**

The treat-to-target (T2T) strategy requires objective biomarkers in inflammatory bowel disease (IBD). Red cell distribution width (RDW) is of interest and routinely available; however, its incremental diagnostic value beyond anemia and inflammatory markers for detecting endoscopic activity (and histologic activity in ulcerative colitis) remains unclear.

**Materials and Methods:**

This retrospective cohort included 1000 IBD patients (ulcerative colitis (UC) = 580, Crohn’s disease (CD) = 420). Objective activity was defined endoscopically (UC: Mayo endoscopic subscore ≥2; CD: SES-CD ≥3), with histologic activity (Nancy Index ≥1) as a prespecified secondary endpoint in UC. Red cell distribution width was measured within a peri-endoscopic window (median 1 day; IQR 0-3). Diagnostic performance was assessed using receiver operating characteristic (ROC) analysis, reporting observed and optimism-corrected area under the curve (AUC). Incremental value over C-reactive protein (CRP) was evaluated using the DeLong method.Multivariable logistic regression was adjusted for anemia, iron-related parameters, inflammatory markers, disease extent, and medications.

**Results:**

Active disease is present in 432 patients (43.2%). The RDW is higher in active vs. inactive disease (15.6 ± 1.8% vs 13.9 ± 1.4%; *P *< .001) and shows good discrimination (observed AUC 0.820; optimism-corrected AUC 0.815). A cut-off of 14.5% yield 78.9% sensitivity and 76.3% specificity. Adding RDW to CRP improve AUC from 0.845 to 0.880 (*P *= .002).Each 1% RDW increase independently predict active disease (adjusted OR 1.65; 95% CI, 1.30-2.09), including non-anemic patients. In UC, RDW increase with histological severity (*P *< .001).

**Conclusion:**

Red cell distribution width independently reflects objective inflammatory activity in IBD and provides incremental value beyond standard markers**, **supporting its role as a low-cost adjunct for T2T monitoring.

**Main Points**

- Red cell distribution width (RDW) is independently associated with objective endoscopic disease activity in a large, real-world cohort of 1000 patients with IBD and with histological activity in ulcerative colitis, even after comprehensive adjustment for anemia, iron-related parameters, inflammatory markers, and medication use.
- Red cell distribution width demonstrates good diagnostic accuracy (optimism-corrected AUC 0.815) for identifying endoscopically active disease, with a clinically practical cut-off value of 14.5%.
- In ulcerative colitis, RDW increases progressively with histological severity, directly linking this routinely available hematological parameter to a key treat-to-target endpoint.
- As an inexpensive and universally available component of the complete blood count, RDW may serve as a pragmatic adjunct to support noninvasive monitoring and prioritize endoscopic assessment in clinical practice.

## Introduction

Inflammatory bowel diseases (IBD), comprising ulcerative colitis (UC) and Crohn’s disease (CD), are chronic immune-mediated disorders characterized by relapsing and remitting gastrointestinal inflammation.^1^ Accurate and objective assessment of disease activity is central to therapeutic decision-making, particularly within contemporary treat-to-target (T2T) strategies, which emphasize predefined endoscopic and, increasingly, histological healing targets rather than symptom control alone.^2^ Current monitoring tools include clinical indices (e.g., partial Mayo score and Harvey–Bradshaw Index), biochemical markers such as C-reactive protein (CRP) and fecal calprotectin, and standardized endoscopic and histologic scoring systems, including the Mayo endoscopic subscore, the Ulcerative Colitis Endoscopic Index of Severity (UCEIS), the Simple Endoscopic Score for Crohn’s Disease (SES-CD), and the Nancy Histologic Index.^3^^-^^5^

Although endoscopy remains the gold standard for evaluating mucosal inflammation, its invasiveness, cost, need for bowel preparation, and impact on patient burden limit its feasibility for frequent monitoring.^6^ Fecal calprotectin is a validated noninvasive biomarker; however, its accuracy varies by disease location, and preanalytical limitations, particularly sample collection issues, may hinder its routine use in real-world clinical practice.^7^ These constraints highlight the need for simple, reproducible, and universally accessible blood-based markers that can support objective assessment of disease activity and reduce reliance on repeated endoscopy.

Red cell distribution width (RDW), a parameter routinely reported in the complete blood count, reflects the heterogeneity of erythrocyte size. Traditionally used in the evaluation of anemia,^8^ RDW has been more recently recognized as a nonspecific surrogate indicator of systemic inflammation. Pro-inflammatory cytokines, particularly interleukin-6 and tumor necrosis factor-*α*, may impair erythropoiesis, promote hepcidin-mediated iron restriction, and induce oxidative injury in erythrocytes, leading to anisocytosis.^9^^,^^10^ Elevated RDW values have been observed across a range of inflammatory and chronic conditions.^11^ In IBD, higher RDW levels have been associated with greater endoscopic severity, poorer response to biologic therapy, increased rates of hospitalization, and a higher systemic inflammatory burden, as supported by both individual studies and a meta-analysis of prior evidence.^12^^-^^17^ The ongoing search for accessible biomarkers is further underscored by recent studies evaluating routine hematological indices in inflammatory bowel disease.^18^ This need is also reflected by contemporary real-world data from Türkiye, including a multicenter 3-decade analysis of IBD (1993-2024).^19^

Despite these observations, several important gaps remain in the existing evidence, which limit the translation of RDW into routine clinical practice. First, most prior individual studies included relatively small, heterogeneous cohorts (median sample size ≈287) with inconsistent definitions of disease activity and inadequate adjustment for confounding factors such as anemia, iron-related parameters, and concurrent medication use—all of which can significantly influence RDW values.^16^ Second, although relationships with clinical or endoscopic activity have been described, robust data evaluating RDW in direct relation to histologic inflammation—an increasingly important target within treat-to-target strategies—remain scarce.^2^^,^^20^ Third, no large real-world study has systematically compared the diagnostic performance of RDW with established biomarkers, such as CRP and fecal calprotectin, or formally assessed whether RDW provides incremental diagnostic value beyond anemia- and iron-related indices. Finally, potential phenotype-specific differences in RDW behavior between UC and CD, given their distinct inflammatory patterns and nutritional consequences, have not been thoroughly explored in adequately powered subgroup analyses.

To address these gaps, a large, well-characterized, single-center cohort study of 1000 patients with IBD was conducted, integrating standardized clinical, endoscopic, and histological assessments. Rather than merely confirming prior associations, the current study aimed to overcome key methodological limitations through its large sample size, use of stringent and objective outcome definitions, comprehensive adjustment for confounders (including anemia, iron status, and medications), and direct comparison with standard biomarkers. The primary aim was to determine whether RDW independently predicts objective disease activity—with a specific focus on endoscopic and histologic inflammation—after rigorous adjustment for anemia-related and other relevant confounders. The secondary aims were to evaluate the diagnostic accuracy of RDW relative to CRP and fecal calprotectin, to formally test its incremental value, and to explore disease-specific patterns in UC and CD. It was hypothesized that RDW may serve as a pragmatic adjunct biomarker within T2T-based care algorithms, owing to its universal availability, low cost, and suitability for repeated measurement.

## Materials and Methods

### Study Design and Setting

This retrospective observational cohort study was conducted at a high-volume tertiary inflammatory bowel disease (IBD) referral center in Türkiye. Clinical, laboratory, endoscopic, and histological data were obtained from the institutional IBD registry. The study methodology adhered to the Strengthening the Reporting of Observational Studies in Epidemiology (STROBE) guidelines and, for its diagnostic accuracy components, the Standards for Reporting Diagnostic Accuracy Studies (STARD) 2015 guideline. A completed STARD 2015 checklist is provided as Supplementary Table 1.^21^

Ethical approval was obtained from Kayseri City Hospital Clinical Research Ethics Committee (Approval No: 2025/289; Date: January 7, 2025). The requirement for written informed consent was waived as the study used anonymized registry data.

### Study Population

**Figure 1.**
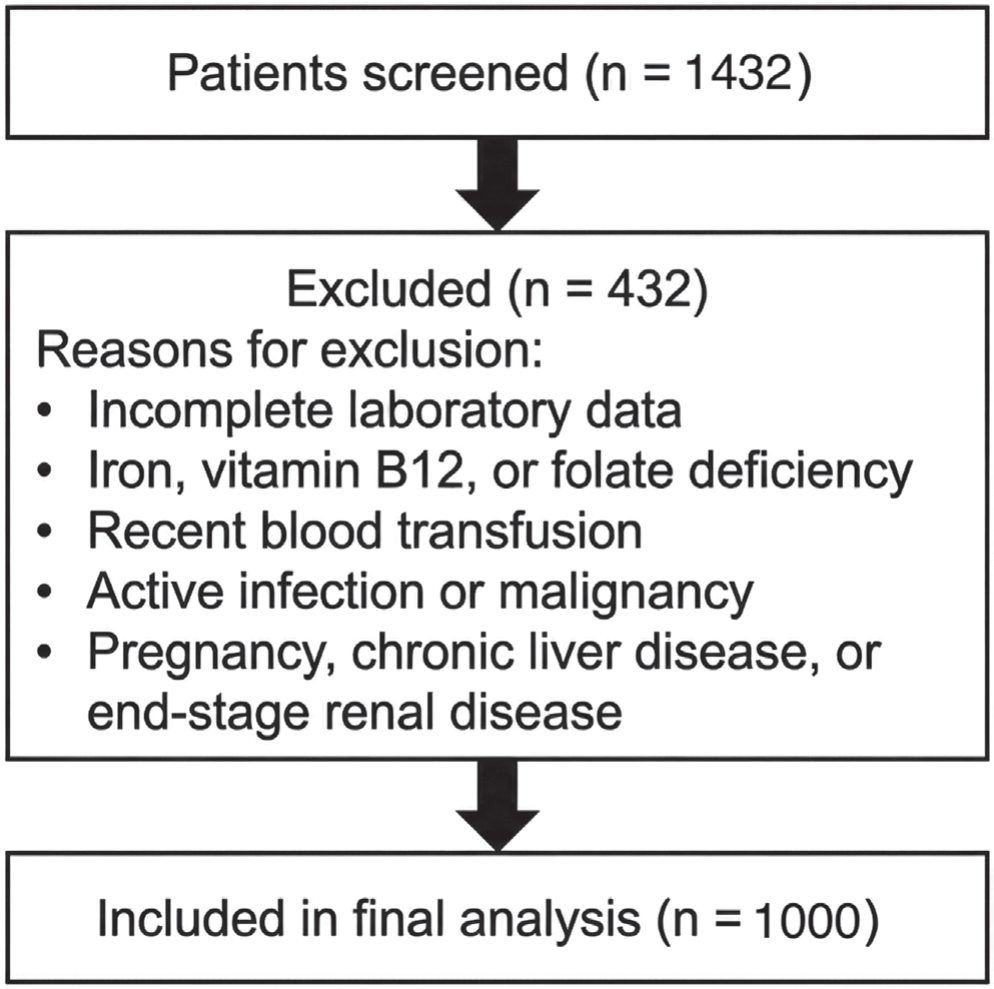
STARD-compliant participant flow diagram of the study cohort. From an initial screen of 1432 patients, 1000 met all inclusion and exclusion criteria and were included in the final analysis.

Consecutive adult patients (≥18 years) with a confirmed diagnosis of ulcerative colitis or Crohn’s disease according to the European Crohn’s and Colitis Organisation (ECCO) criteria were screened between January 2020 and December 2024. Diagnosis was established in accordance with the ECCO consensus statements for ulcerative colitis and Crohn’s disease.^22^^,^^23^ To ensure temporal proximity and minimize confounding from changes in clinical status, all laboratory tests and the index endoscopy were required to be performed during the same clinical evaluation episode. The maximum permitted interval between blood draw and endoscopy was 7 days, with a median (interquartile range, IQR) interval of 1 (0-3) day. From an initial screen of 1432 patients, 1000 met all inclusion and exclusion criteria. A STARD-compliant participant flow diagram is provided in Figure 1.

### Inclusion Criteria

- Availability of complete laboratory data (RDW, hemoglobin, mean corpuscular volume (MCV), C-reactive protein (CRP), erythrocyte sedimentation rate (ESR), albumin, ferritin).
- A complete ileocolonoscopy or colonoscopy (for UC) with documented endoscopic activity scoring performed within the defined peri-laboratory window.
- For UC, availability of colonic biopsy results assessed using the Nancy Histological Index.^5^
- Documented disease extent according to the Montreal classification.^22^^,^^23^

### Exclusion Criteria

- Hematologic disorders affecting red cell indices (e.g., thalassemia, hereditary spherocytosis).
- Significant iron, vitamin B_12_, or folate deficiency at the time of assessment (ferritin <30 ng/mL; vitamin B_12_ <200 pg/mL; folate <3 ng/mL).^24^
- Blood transfusion within the preceding 3 months.
- Active infection (clinically suspected or defined as CRP >50 mg/L with concomitant leukocytosis) or active malignancy.
- Pregnancy, chronic liver disease, or end-stage renal disease.

### Data Collection

Demographic and clinical variables included age, sex, disease duration, Montreal classification, smoking status, and prior IBD-related surgery. Disease extent was classified according to the Montreal classification and used as a covariate in multivariable analyses. Medications at the time of assessment were recorded in detail: 5-aminosalicylates, corticosteroids (systemic and local), immunomodulators (thiopurines, methotrexate), biologic agents (anti–tumor necrosis factor, anti-integrin, anti–interleukin-12/23), and Janus kinase inhibitors.

Fecal calprotectin (FC) was measured using a quantitative immunoassay (Bühlmann fCAL® turbo) on a single stool sample collected within the same peri-endoscopic window (≤7 days), with an active disease cut-off of ≥150 µg/g. Fecal calprotectin data were available for a subset of patients (n = 602, 60.2%); analyses involving FC were considered exploratory, and baseline characteristics of this subgroup were compared with the overall cohort to assess potential selection bias. Fecal calprotectin was unavailable in the remaining patients (39.8%), primarily because, in this real-world retrospective cohort, FC was not ordered at every peri-endoscopic encounter. In addition, stool submission and processing were patient dependent; therefore, missed collections and logistical constraints within the predefined time window were common.

### Laboratory Measurements

All blood samples were processed within 2 hours. Red Cell Distribution Width–Coefficient of Variation (RDW-CV) (%) was measured using a Sysmex XN-1000™ analyzer.^25^ C-reactive protein was measured via immunoturbidimetric assay (Abbott Architect™). Erythrocyte sedimentation rate was determined using the Westergren method.^26^

### Disease Activity Assessment

The primary outcome was endoscopically active disease, defined using prespecified criteria for each phenotype (UC: Mayo endoscopic subscore ≥2; CD: SES-CD ≥3).

Ulcerative colitis: Active disease was defined by a Mayo endoscopic subscore ≥2.^27^

In prespecified sensitivity analyses, we additionally evaluated:

1. Histologic activity alone (Nancy Index ≥1) and
2. Combined objective activity (endoscopic (Mayo ≥2) and/or histologic (Nancy ≥1)), irrespective of clinical symptom scores.

Crohn’s disease: Active disease was defined by a Simple Endoscopic Score for Crohn’s Disease (SES-CD) ≥3.^4^

Clinical indices (partial Mayo score for UC, Harvey–Bradshaw Index (HBI)^28^ for CD) were recorded but not used to define the primary outcome.

All endoscopic procedures were performed by expert IBD endoscopists. A second blinded-reader reviewed a random 10% sample for quality assurance (interobserver agreement: κ = 0.85 for UCEIS and 0.79 for SES-CD).

### Statistical Analysis

The normality of continuous variables was assessed using the Shapiro–Wilk test. Data are presented as mean ± standard deviation (SD) for normally distributed variables or median (interquartile range, IQR) for non-normally distributed variables.

An a priori power analysis indicated that 968 patients would provide 90% power (*α* = .05) to detect a correlation of ρ = 0.32 between RDW and endoscopic activity.

Primary analyses included:

- Spearman correlation between RDW and disease activity indices.
- Receiver operating characteristic (ROC) analysis to evaluate RDW’s diagnostic accuracy using endoscopic activity as the reference standard. For the primary RDW cut-off, sensitivity, specificity, positive and negative predictive values (PPV, NPV), and positive and negative likelihood ratios (LR+, LR−) were calculated.
- Internal validation was performed using 1000 bootstrap samples to calculate optimism-corrected AUC estimates.
- To evaluate incremental value, the AUC of a model containing only standard biomarkers (CRP, ESR) was compared to a model incorporating RDW using the DeLong test.^29^
- Multivariable logistic regression assessed the independent association between RDW (per 1% increase) and objectively active disease.^30^

Base model covariates included age, sex, hemoglobin, MCV, albumin, and disease extent (Montreal classification). A secondary, fully adjusted model additionally included CRP, ESR, and current use of corticosteroids and biologic agents.

Subgroup and sensitivity analyses included:

- Separate analyses for UC and CD.
- Sensitivity analysis restricted to non-anemic patients (anemia defined per WHO criteria as hemoglobin <13 g/dL for men and <12 g/dL for women).^24^
- For UC, ordinal logistic regression evaluation for the association between RDW and Nancy histologic categories.
- Analyses for the FC subgroup were performed separately, with a comparison of baseline characteristics to the overall cohort to assess potential selection bias.

All analyses were performed using SPSS v27.0 (IBM SPSS Corp.; Armonk, NY, USA) and R v4.2.1 (R Foundation for Statistical Computing; Vienna, Austria). A 2-sided *P*-value < .05 was considered statistically significant.

## Results

A total of 1000 patients with inflammatory bowel disease were included in the final analysis (Figure 1). The cohort comprised 580 patients with ulcerative colitis (UC: 58.0%) and 420 with Crohn’s disease (CD: 42.0%). The mean age was 42.8 ± 14.6 years, and 54.3% were male. The median disease duration was 6.2 years (interquartile range (IQR), 3.1-10.5). Based on the primary endoscopic outcome definition, 432 patients (43.2%) had objectively active disease (UC, 260/580 (44.8%); CD, 172/420 (41.0%)).

Baseline characteristics according to disease activity status are summarized in Table 1. Compared with patients in remission, those with active disease had significantly lower hemoglobin (12.2 ± 1.8 vs. 13.4 ± 1.6 g/dL; *P* < .001) and albumin (3.9 ± 0.5 vs. 4.3 ± 0.4 g/dL; *P* < .001) levels, and markedly higher inflammatory markers (CRP and ESR). Red cell distribution width was significantly higher in active disease (15.6 ± 1.8% vs. 13.9 ± 1.4%; *P* < .001). This finding was consistent in both UC (15.4 ± 1.7% vs. 13.8 ± 1.3%; *P* < .001) and CD (15.8 ± 1.9% vs. 14.0 ± 1.5%; *P* < .001). A detailed distribution of disease extent (Montreal classification) and current medication use is provided in Supplementary Table 2. As expected, extensive colitis (E3) in UC and ileocolonic involvement (L3) in CD were more frequent in active cases. Current use of corticosteroids (32.4% vs. 11.3%; *P* < .001) and biologic agents (45.6% vs. 38.2%; *P* = .02) was also higher in the active disease group.

### Correlation of Red Cell Distribution Width with Inflammatory Markers and Disease Indices

Red cell distribution width showed a moderate positive correlation with CRP (*r* = 0.52; *P* < .001) and ESR (*r* = 0.49; P < .001), and an inverse correlation with hemoglobin (*r* = −0.31; *P* < .001). RDW also correlated negatively with serum albumin (*r* = −0.41; *P* < .001), ferritin (*r* = −0.25; *P* < .001), and transferrin saturation (*r* = −0.29; *P* < .001). Consistent with these correlations, serum albumin (3.9 ± 0.5 vs. 4.3 ± 0.4 g/dL), ferritin (65 (IQR 30-120) vs. 85 (IQR 45-150) ng/mL), and transferrin saturation (18 ± 8% vs. 24 ± 9%) levels were all significantly lower in patients with active disease (all *P* < .05; see Table 1).

In UC, RDW correlated significantly with the Mayo endoscopic subscore (*r* = 0.47; *P* < .001). In CD, RDW correlated with the Harvey–Bradshaw Index (*r* = 0.44; *P* < .001) and the SES-CD (*r* = 0.46; *P* < .001).

### Diagnostic Performance of Red Cell Distribution Width

**Figure 2.**
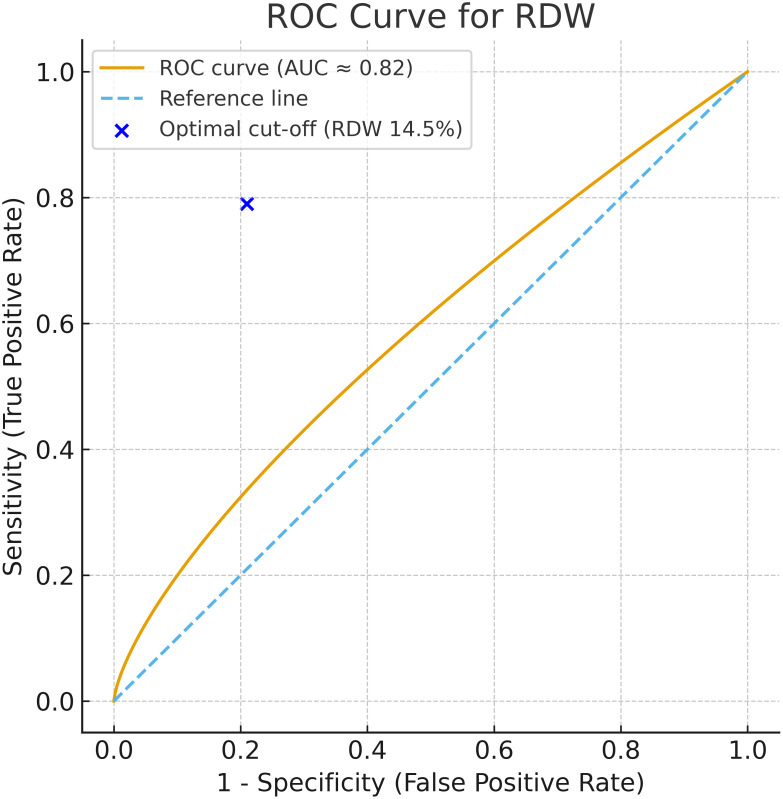
Receiver operating characteristic (ROC) curve of red cell distribution width (RDW) for discriminating active versus inactive inflammatory bowel disease (IBD). The curve demonstrates an observed area under the curve (AUC) of 0.82 (95% CI, 0.80-0.85). The optimal cut-off (14.5%) corresponds to 78.9% sensitivity and 76.3% specificity.

Receiver operating characteristic analysis demonstrated good diagnostic accuracy for RDW in distinguishing endoscopically active disease from inactive (Figure 2), with an observed AUC of 0.82 (95% CI, 0.80-0.85). The optimism-corrected AUC based on 1000 bootstrap samples was 0.815 (95% CI, 0.790-0.840). The optimal cut-off identified by the Youden Index was 14.5%, corresponding to a sensitivity of 78.9% and a specificity of 76.3% (Table 2). This cut-off yielded a positive predictive value (PPV) of 75.2%, a negative predictive value (NPV) of 79.8%, a positive likelihood ratio (LR+) of 3.33, and a negative likelihood ratio (LR−) of 0.28.

The diagnostic performance was similar in UC (AUC 0.810, optimal cut-off 14.4%) and CD (AUC 0.830, optimal cut-off 14.6%) (Table 2). The AUC for CRP alone (cut-off 5 mg/L) was 0.845 (95% CI, 0.820-0.870). The combination of RDW and CRP in a logistic regression model significantly improved the AUC to 0.880 (95% CI, 0.860-0.900; P = .002 by DeLong test).

In the fecal calprotectin subgroup (n = 602), median fecal calprotectin levels were significantly higher in patients with active disease compared with those in remission (320 (IQR 180-650) µg/g vs. 85 (IQR 45-150) µg/g; *P* < .001; Supplementary Table 2). This subgroup was representative of the overall cohort, with no significant differences in key baseline characteristics (all *P* > .1). In this subgroup, fecal calprotectin using a cut-off of ≥150 µg/g demonstrated excellent diagnostic performance, with an AUC of 0.875 (95% CI, 0.847-0.903).

### Sensitivity Analyses Using Alternative Outcome Definitions

When using alternative prespecified definitions in UC, RDW (cut-off 14.5%) demonstrated an AUC of 0.800 (95% CI, 0.765-0.835) for identifying histologic activity alone (Nancy Index ≥1), and an AUC of 0.815 (95% CI, 0.782-0.848) for identifying combined objective activity (endoscopic and/or histologic).

### Multivariable Analysis

In multivariable logistic regression, each 1% increase in RDW remained independently associated with active disease after adjustment for age, sex, hemoglobin, MCV, albumin, and disease extent (adjusted OR = 2.41; 95% CI, 1.89-3.07; *P* < .001). In the fully adjusted model, which additionally included CRP, ESR, and current use of corticosteroids and biologic agents, RDW retained a significant, albeit attenuated, association (adjusted OR = 1.65; 95% CI, 1.30-2.09; *P* < .001). Complete regression results, including all covariates, are presented in Supplementary Table 3.

A sensitivity analysis restricted to non-anemic patients (n = 712) confirmed the independent predictive value of RDW (adjusted OR = 2.05; 95% CI, 1.52-2.78; *P* < .001).

### Histologic and Endoscopic Severity Stratification in Ulcerative Colitis

Among patients with UC, RDW increased progressively across both endoscopic and histologic severity grades (Table 3). The proportion of patients with RDW >14.5% increased from 18.1% in endoscopic remission (Mayo 0) to 82.2% in severe endoscopic activity (Mayo 3), and from 22.0% in histologic remission (Nancy 0) to 80.0% in severe histologic activity (Nancy 3). Ordinal regression confirmed that higher RDW independently predicted more severe Nancy histologic categories (OR per 1% RDW increase = 1.48; 95% CI, 1.32-1.66; *P* < .001).

## Discussion

In this large, well-phenotyped single-center cohort of 1000 patients, RDW demonstrates a consistent and independent association with objective inflammatory activity in both UC and CD. The current study builds on prior evidence by confirming this relationship in a substantially larger cohort, employing stringent endoscopic and histological outcome definitions, performing comprehensive adjustment for confounders (including anemia, iron status, disease extent, and medication use), and formally evaluating the incremental diagnostic value of RDW. By addressing key methodological limitations of prior studies, these findings strengthen the evidence supporting RDW as a marker of objective inflammatory burden in IBD rather than a nonspecific correlate of anemia. The observed discriminatory performance of RDW (AUC ~0.82) is comparable to that reported in previous meta-analyses and smaller studies, which suggestes RDW’s potential utility but are limited by sample size and methodological heterogeneity.^16^^,^^17^ Importantly, the analysis confirms that RDW provides diagnostic value beyond its traditional association with anemia, as evidenced by its persistent independent association in multivariable models and among non-anemic patients.

The findings are consistent with the proposed biological plausibility of RDW as a marker of systemic inflammation. Pro-inflammatory cytokines, notably interleukin-6, can induce hepcidin-mediated iron restriction and impair erythropoiesis, leading to increased heterogeneity in erythrocyte size.^9^^,^^10^ The graded association between RDW and Nancy histological grades in UC provides supportive clinical evidence linking this routinely available hematological parameter to microscopic mucosal inflammation, an established treatment target in contemporary IBD management.^5^ This stepwise relationship across histologic severity categories further supports the relevance of RDW within treat-to-target frameworks that increasingly emphasize objective mucosal healing.^2^ The independent association of RDW with endoscopic activity after adjustment for CRP and ESR suggests that RDW may reflect aspects of inflammatory burden not fully captured by conventional acute-phase reactants, without implying distinct mechanistic pathways.^7^^,^^11^

From a clinical perspective, RDW offers several practical advantages as a universally available, low-cost component of the complete blood count. Its potential role within multiparameter assessment frameworks aligns with the growing interest in routine hematological indices for IBD monitoring.^18^ However, RDW should be interpreted as an adjunct rather than a standalone test: at the pragmatic cut-off of 14.5%, sensitivity (78.9%) and specificity (76.3%) remained below 80%, with moderate PPV (75.2%) and NPV (79.8%). Its incremental value was most evident when combined with CRP, improving discrimination from an AUC of 0.845 (CRP alone) to 0.880 (RDW+CRP; *P* = .002 by DeLong test), supporting its use within multimarker, noninvasive assessment strategies. The reported likelihood ratios (LR+ 3.33; LR− 0.28) provide clinicians with quantitative measures that may aid interpretation in individual patients. In routine practice, persistently normal RDW values in conjunction with other reassuring clinical and laboratory findings may contribute to clinical reassurance, whereas increasing RDW values may prompt consideration of closer follow-up or additional evaluation, rather than serving as a standalone trigger for intervention.

Phenotype-specific patterns are also observed. The slightly higher optimal RDW cut-off identified in CD compared with UC may be related to the combined effects of systemic inflammation, small-bowel involvement, and nutritional disturbances more frequently encountered in CD; however, the observation of this study precludes confirmation of distinct mechanistic explanations. In UC, the strong association between RDW and both endoscopic and histologic severity underscores its close relationship with colonic inflammatory burden.

In contrast, histologic stratification is not performed in CD. This is a deliberate methodological choice rather than a limitation. Histologic assessment in CD is inherently constrained by the patchy and transmural nature of inflammation, and currently, there is no universally accepted, routinely applied histologic severity index for CD in clinical practice or within treat-to-target strategies. Accordingly, and in line with current ECCO recommendations, endoscopic activity assessed by the SES-CD is considered the most appropriate objective outcome for CD in the present study.^22^^,^^23^

Several limitations of the current study should be acknowledged. The retrospective, single-center design may limit generalizability and does not allow causal inference. The cross-sectional analysis preclude assessment of longitudinal changes in RDW in relation to treatment response or relapse, which would be particularly relevant for treat-to-target strategies. Fecal calprotectin is not available for all patients, although the analyzed subgroup is representative of the overall cohort. In addition, analytical variability between RDW-CV and RDW-SD measurements highlights the need for standardization before broader clinical application. Nevertheless, the large sample size, rigorous outcome definitions, and extensive adjustment for relevant confounders strengthen the validity of the findings.

Prospective, multicenter studies are warranted to validate these results, assess the temporal dynamics of RDW, and explore its integration into composite predictive models alongside established biomarkers. Emerging biomarkers reported in recent studies may further enrich such multimarker frameworks, including newly proposed markers in ulcerative colitis such as serum histone H4.^31^^,32^ Exploratory computational or machine-learning–based approaches incorporating RDW may be of interest, but their clinical utility will require formal validation.^33^

In conclusion, RDW is an accessible and inexpensive laboratory parameter that is independently associated with endoscopic disease activity in both UC and CD and correlates with histological severity in UC. Its association with objective inflammation persists after adjustment for anemia-related, nutritional, and inflammatory factors, supporting RDW as an adjunctive marker rather than a surrogate for hemoglobin status. Although not intended to replace endoscopy or established biomarkers such as fecal calprotectin, RDW may provide complementary information within noninvasive monitoring strategies and may support treat-to-target decision-making when interpreted alongside established clinical and laboratory measures.

## Supplementary Materials

Supplementary Material

## Figures and Tables

**Table 1.** Baseline Demographic and Clinical Characteristics of Patients According to Objective Disease Activity Status

**Variable**	**Inactive Disease (n = 568)**	**Active Disease (n = 432)**	***P***
Age (years)	43.1 ± 14.4	42.4 ± 14.8	.420
Male, n (%)	306 (53.9)	237 (54.9)	.760
Disease duration (years)	6.1 (3.0-10.4)	6.4 (3.2-10.7)	.380
Hemoglobin (g/dL)	13.4 ± 1.6	12.2 ± 1.8	<.001
Albumin (g/dL)	4.3 ± 0.4	3.9 ± 0.5	<.001
Ferritin (ng/mL)	85 (45-150)	65 (30-120)	.021
Transferrin saturation (%)	24 ± 9	18 ± 8	.012
RDW (%)	13.9 ± 1.4	15.6 ± 1.8	<.001
CRP (mg/L)	5.8 (3.0-10.7)	18.4 (9.1-37.5)	<.001
ESR (mm/h)	14 (8-24)	32 (18-48)	<.001

Values are expressed as mean ± standard deviation or median (IQR), unless otherwise indicated.

CRP, C-reactive protein; ESR, erythrocyte sedimentation rate; IQR, interquartile range; RDW, red cell distribution width.

**Table 2.** Diagnostic Performance of RDW for Detecting Objectively Active Disease

**Subgroup**	**AUC (95% CI)**	**Optimal cut-off (%)**	**Sensitivity (%)**	**Specificity (%)**
Overall	0.820 (0.800-0.850)	14.5	78.9	76.3
UC	0.810 (0.780-0.840)	14.4	77.3	75.6
CD	0.830 (0.800-0.860)	14.6	79.7	77.1

PPV, NPV, LR+ and LR− values are reported in the results section.

AUC, area under the curve; CD, Crohn’s disease; CI, confidence interval; UC, ulcerative colitis.

**Table 3.** Distribution of RDW According to Endoscopic and Histologic Severity in Ulcerative Colitis

RDW According to Endoscopic Severity (Mayo Endoscopic Subscore)			
Mayo endoscopic subscore	n	RDW (%) mean ± SD	RDW >14.5%, n (%)
0 (Remission)	122	13.6 ± 1.2	22 (18.1)
1 (Mild)	198	14.2 ± 1.3	61 (30.8)
2 (Moderate)	167	15.3 ± 1.5	104 (62.3)
3 (Severe)	93	16.4 ± 1.8	76 (82.2)
P for trend		**< 0.001**	
RDW According To Histologic Severity (Nancy Histological Index)			
Nancy histologic grade	n	RDW (%) mean ± SD	RDW >14.5%, n (%)
0 (Remission)	150	13.7 ± 1.3	33 (22.0)
1 (Mild)	186	14.4 ± 1.4	58 (31.2)
2 (Moderate)	149	15.2 ± 1.6	91 (61.1)
3 (Severe)	95	16.1 ± 1.9	76 (80.0)
P for trend		**< 0.001**	

Values are presented as mean ± standard deviation or number (%), as appropriate. Endoscopic severity was assessed using the Mayo endoscopic subscore. Histologic severity was assessed using the Nancy Histological Index. P for trend was calculated using ordinal regression analysis.

RDW, red cell distribution width.

## Data Availability

The data that support the findings of this study are available on request from the corresponding author.
