# Supplementary material for: Red Cell Distribution Width as an Independent Marker of Objective Disease Activity in Ulcerative Colitis and Crohn’s Disease: A Large Real-World Cohort of 1000 Patients
Source: Turk J Gastroenterol. 2026 Apr 8;37(6):693–701. doi: 10.5152/tjg.2026.25788 (PMC13247870; doi:10.5152/tjg.2026.25788)
Supplement: Supplementary Material [file supplementary_material.pdf]

Supplementary Table 1. STARD checklist for reporting of studies of diagnostic accuracy

Section and Topic	Item #		On page #
TITLE/ABSTRACT/ KEYWORDS	1	Identify the article as a study of diagnostic accuracy (recommend MeSH heading 'sensitivity and specificity').	Title; Abstract
INTRODUCTION	2	State the research questions or study aims, such as estimating diagnostic accuracy or comparing accuracy between tests or across participant groups.	Introduction (final paragraph)
METHODS			
Participants	3	The study population: The inclusion and exclusion criteria, setting and locations where data were collected.	Methods – Study Population; Inclusion Criteria; Exclusion Criteria
	4	Participant recruitment: Was recruitment based on presenting symptoms, results from previous tests, or the fact that the participants had received the index tests or the reference standard?	Methods – Study Population
	5	Participant sampling: Was the study population a consecutive series of participants defined by the selection criteria in item 3 and 4? If not, specify how participants were further selected.	Methods – Study Population
	6	Data collection: Was data collection planned before the index test and reference standard were performed (prospective study) or after (retrospective study)?	Methods – Study Design and Setting
Test methods	7	The reference standard and its rationale.	Methods – Disease Activity Assessment
	8	Technical specifications of material and methods involved including how and when measurements were taken, and/or cite references for index tests and reference standard.	Methods – Laboratory Measurements; Disease Activity Assessment
	9	Definition of and rationale for the units, cut-offs and/or categories of the results of the index tests and the reference standard.	Methods – Statistical Analysis
	10	The number, training and expertise of the persons executing and reading the index tests and the reference standard.	Methods – Disease Activity Assessment
	11	Whether or not the readers of the index tests and reference standard were blind (masked) to the results of the other test and describe any other clinical information available to the readers.	Methods – Disease Activity Assessment
Statistical methods	12	Methods for calculating or comparing measures of diagnostic accuracy, and the statistical methods used to quantify uncertainty (e.g. 95% confidence intervals).	Methods – Statistical Analysis
	13	Methods for calculating test reproducibility, if done.	Not applicable
RESULTS			
Participants	14	When study was performed, including beginning and end dates of recruitment.	Methods – Study Population
	15	Clinical and demographic characteristics of the study population (at least information on age, gender, spectrum of presenting symptoms).	Results – Baseline Characteristics; Table 1
	16	The number of participants satisfying the criteria for inclusion who did or did not undergo the index tests and/or the reference standard; describe why participants failed to undergo either test (a flow diagram is strongly recommended).	Figure 1; Methods – Study Population
Test results	17	Time-interval between the index tests and the reference standard, and any treatment administered in between.	Methods – Study Population
	18	Distribution of severity of disease (define criteria) in those with the target condition; other diagnoses in participants without the target condition.	Results – Disease Severity Stratification; Table 3
	19	A cross tabulation of the results of the index tests (including indeterminate and missing results) by the results of the reference standard; for continuous results, the distribution of the test results by the results of the reference standard.	Results – Diagnostic Performance; Tables 2 and 3

(*Continued*)

Supplementary Table 1. STARD checklist for reporting of studies of diagnostic accuracy (Continued)

Section and Topic	Item #		On page #
	20	Any adverse events from performing the index tests or the reference standard.	Not applicable
Estimates	21	Estimates of diagnostic accuracy and measures of statistical uncertainty (e.g. 95% confidence intervals).	Results – Diagnostic Performance
	22	How indeterminate results, missing data and outliers of the index tests were handled.	Methods – Data Collection; Results (FC subgroup)
	23	Estimates of variability of diagnostic accuracy between subgroups of participants, readers or centers, if done.	Results – Subgroup and Sensitivity Analyses
	24	Estimates of test reproducibility, if done.	Not applicable
DISCUSSION	25	Discuss the clinical applicability of the study findings.	Discussion; Conclusions

Supplementary Table 2. Disease extent, current medication use, and fecal calprotectin levels according to objective disease activity status

Disease Extent (Montreal Classification) – Ulcerative Colitis			
Variable	Inactive (n = 320)	Active (n = 260)	***P***
E1 (Proctitis), n (%)	83 (25.94)	52 (20.00)	.094
E2 (Left-sided colitis), n (%)	141 (44.06)	103 (39.62)	.273
E3 (Extensive colitis), n (%)	96 (30.00)	105 (40.38)	.009
**Disease Extent (Montreal Classification) – Crohn's Disease**			
**Variable**	**Inactive (n = 248)**	**Active (n = 172)**	**P**
L1 (Ileal), n (%)	92 (37.10)	52 (30.23)	.148
L2 (Colonic), n (%)	72 (29.03)	41 (23.84)	.242
L3 (Ileocolonic), n (%)	84 (33.87)	79 (45.93)	.012
**Current Medication Use at the Time of Assessment (Entire Cohort, N = 1000)**			
**Medication**	**Inactive disease (n = 568)**	**Active disease (n = 432)**	**P**
5-aminosalicylates, n (%)	392 (69.0)	281 (65.0)	.18
Corticosteroids (any), n (%)	64 (11.3)	140 (32.4)	<.001
Immunomodulators (thiopurines / MTX), n (%)	214 (37.7)	176 (40.7)	.34
Biologic agents (any), n (%)	217 (38.2)	197 (45.6)	.02
Anti-TNF agents, n (%)	132 (23.2)	118 (27.3)	.14
Anti-integrin agents, n (%)	46 (8.1)	41 (9.5)	.44
Anti–IL-12/23 agents, n (%)	39 (6.9)	38 (8.8)	.26
JAK inhibitors, n (%)	21 (3.7)	18 (4.2)	.71
**Fecal Calprotectin Levels According to Objective Disease Activity in the Subgroup with Available Data (N = 602)**			
**Variable**	**Inactive disease (n = 340)**	**Active disease (n = 262)**	**P**
Fecal calprotectin (μg/g), median (IQR)	85 (45–150)	320 (180–650)	<.001*

Values are presented as number (%) or median (interquartile range), as appropriate.
Disease extent was classified according to the Montreal classification.
Objective disease activity was defined by endoscopic criteria as described in the Methods (UC: Mayo endoscopic subscore ≥2; CD: SES-CD ≥3). Fecal calprotectin was measured using a quantitative immunoassay (Bühlmann fCAL® turbo) on stool samples collected within ≤7 days of the index endoscopy. The distribution of patients in the fecal calprotectin subgroup (340 inactive, 262 active) was proportional to that of the overall cohort (568 inactive, 432 active), suggesting limited potential for selection bias.
IL, interleukin; IQR, interquartile range; JAK, janus kinase; MTX, methotrexate; TNF, tumor necrosis factor.
*P-value calculated using the Mann–Whitney U-test.

Supplementary Table 3. Multivariable logistic regression analyses for objectively active disease

Base Multivariable Model			
Variable	Adjusted OR	95% CI	***P***
RDW (per 1% increase)	2.41	1.89–3.07	<.001
Age (per year)	0.99	0.98–1.01	.42
Male sex	1.04	0.81–1.34	.76
Hemoglobin (per g/dL)	0.82	0.74–0.91	<.001
Mean corpuscular volume (per fL)	0.97	0.95–0.99	.01
Albumin (per g/dL)	0.54	0.44–0.67	<.001
Disease extent (Montreal classification)*	—	—	<.01
**Fully Adjusted Multivariable Model**			
**Variable**	**Adjusted OR**	**95% CI**	**P**
RDW (per 1% increase)	1.65	1.30–2.09	<.001
Age (per year)	0.99	0.98–1.01	.45
Male sex	1.02	0.79–1.32	.88
Hemoglobin (per g/dL)	0.88	0.79–0.98	.02
Mean corpuscular volume (per fL)	0.98	0.96–1.00	.08
Albumin (per g/dL)	0.61	0.50–0.75	<.001
C-reactive protein (per mg/L)	1.03	1.02–1.04	<.001
Erythrocyte sedimentation rate (per mm/h)	1.01	1.00–1.02	.01
Corticosteroid use (yes vs no)	2.34	1.72–3.18	<.001
Biologic agent use (yes vs no)	1.42	1.08–1.88	.01
Disease extent (Montreal classification)*	—	—	<.01

Objective disease activity was defined by endoscopic criteria (UC: Mayo endoscopic subscore ≥2; CD: SES-CD ≥3). Odds ratios (ORs) are adjusted for all variables listed in each model. RDW was modeled as a continuous variable per 1% increase. Disease extent was entered as a categorical covariate according to the Montreal classification.
RDW, red cell distribution width.
